# Evaluation of Optical and Acoustical Properties of Ba_1−x_Sr_x_TiO_3_ Thin Film Material Library via Conjugation of Picosecond Laser Ultrasonics with X-ray Diffraction, Energy Dispersive Spectroscopy, Electron Probe Micro Analysis, Scanning Electron and Atomic Force Microscopies

**DOI:** 10.3390/nano11113131

**Published:** 2021-11-20

**Authors:** Sathyan Sandeep, Samuel Raetz, Jerome Wolfman, Beatrice Negulescu, Guozhen Liu, Jean-Louis Longuet, Théo Thréard, Vitalyi E. Gusev

**Affiliations:** 1 Laboratoire d’Acoustique de l’Université du Mans (LAUM), UMR 6613, Institut d’Acoustique-Graduate School (IA-GS), CNRS, Le Mans Université, 72085 Le Mans, France; sandeep.sathyan@univ-lemans.fr (S.S.); samuel.raetz@univ-lemans.fr (S.R.); threard.theo@gmail.com (T.T.); 2 Laboratoire GREMAN, UMR CNRS 7347, Université de Tours, INSA CVL, Parc de Grandmont, 37200 Tours, France; wolfman@univ-tours.fr (J.W.); beatrice.negulescu@univ-tours.fr (B.N.); guozhen.liu@hotmail.com (G.L.); 3 CEA, DAM, Le Ripault, 37260 Monts, France; jean-louis.longuet@cea.fr

**Keywords:** picosecond laser ultrasonics, time-domain Brillouin scattering, optoacoustics, acousto-optics, nanoascale imaging, nanomaterials, laterally graded materials library, graded nanofilms and nanocoatings with continuously varying chemical composition, nanocoating characterization techniques

## Abstract

Wide-range continuous spatial variation of the film composition in lateral compositionally graded epitaxial films requires the development of high throughput measurement techniques for their local and non-destructive characterization with the highest possible spatial resolution. Here we report on the first application of the picosecond laser ultrasonics (PLU) technique for the evaluation of acoustical and optical parameters of lateral compositionally graded film, the Ba_1−x_Sr_x_TiO_3_ (0 ≤ x ≤ 1) material library. The film was not dedicatedly prepared for its opto-acousto-optic evaluation by PLU, exhibiting significant lateral variations in thickness and surface roughness. Therefore, the achieved measurements of the sound velocity and of the optical refractive index, and characterization of the surface roughness confirm the robustness of the PLU technique for thin film evaluation. We hope that the first measurements of the acoustical and optical properties of epitaxial grown Ba_1−x_Sr_x_TiO_3_ (0 ≤ x ≤ 1) by PLU technique accomplished here provide the parameters required for more extended predictive design of the phononic, photonic and phoxonic mirrors and cavities with superior properties/functionalities for novel multifunctional nanodevices.

## 1. Introduction

### 1.1. Graded Nanofilms and Nanocoatings with Continuously Varying Chemical Composition

Materials with graded chemical composition have a variety of applications at different spatial scales. Among the most known functionally graded materials are, perhaps, the laterally graded aperiodic crystals (on the basis of mixed Si_1−x_ Ge_x_ crystals) in optical elements for synchrotron radiation [1] and out-of-plane graded band gap semiconductors in solar cells [2]. The applications of the former include radiation path control (diffraction, focusing) in high-resolution X-ray monochromators [1]. The applications of the latter are multiple in solar cells, where the design of an appropriate grading of a larger band gap for recombination and a lower band gap for absorption provides opportunity to separate the mechanisms of carrier recombination and current generation and to improve harvesting of solar energy [2,3].

When the films, including graded films with continuously varying chemical composition, are prepared for particular applications via epitaxial growth, their local physical parameters are, in general, different from those of bulk materials (substrates) of the same chemical composition. This can be caused by different physical factors, in particular by the difference of the mechanical strain field in differently grown films. For example, in the coherent epitaxy, the in-plane parameter of the film is pinned to that of the substrate, while in the completely relaxed films, it is close to its value in bulk material. The incompletely relaxed films could exhibit intermediate strains. Material libraries [4] are prepared for the studies of the physical parameters’ dependence on the material composition to find their values by optimizing the physical property. An example is epitaxial deposition of continuous in-plane-graded Ba_1−x_Sr_x_TiO_3_ (BSTx) films for the dielectric permittivity and loss, and tunability optimization of BSTO capacitors [5] used in microwave and multifunctional devices [6,7]. Out-of-plane graded buffer layers are prepared to achieve the desirable variation of the material out-of-plane lattice constant from the bottom to the top. An example is the improved crystallinity of Ba_0.6_Sr_0.4_TiO_3_ (BST0.4) film grown on strongly latticed mismatched SrTiO_3_ substrate via the deposition of the intermediate epitaxial compositionally graded (ECG) 270-nm thick buffer film of Ba_1−x_Sr_x_TiO_3_ (0 ≤x≤0.6) [8].

The wide-range continuous spatial variations of the film composition require the development of high-throughput measurement techniques to locally and non-destructively characterize the material library with the highest possible spatial resolution. Among the techniques applied until now for the spatial control of the stoichiometry and of the structure of the laterally graded epitaxial films are energy or wavelength dispersive X-ray spectroscopy, high-resolution X-ray diffraction and Raman spectroscopy/microscopy [9,10,11]. However, optical, acoustical and acousto-optical parameters have never been evaluated in vertical or lateral compositionally graded films. Here we report on the first application of the technique of picosecond laser ultrasonics (PLU) [12,13,14] for the evaluation of the optical refractive index n and of the longitudinal acoustic velocity v in the material library.

A continuous composition thin film of BSTx was deposited over an opaque optoacoustic thin film transducer—required for the application of PLU in transparent films/coatings—by combinatorial pulsed laser deposition (CPLD) on a single substrate [15]. The complete BSTx solid solution could then be studied by PLU on a unique sample. This way, the acoustical and optical parameters, extracted from the acoustically induced changes in the ultrafast pump-probe transient optical reflectivity as a function of local chemical composition, were preserved from run-to-run variation that might occur when successive samples with different chemical compositions are sequentially prepared.

The dependence n=n(x) in BSTx films has not been evaluated earlier, although the spectrophotometry and spectroscopic reflectometry have been applied for the evaluation of in-plane graded amorphous [16] and out-of-plane graded [17] polycrystalline films, respectively. The dependence n=n(x) has only been measured in the non-graded, i.e., compositionally homogeneous, coatings prepared by sol-gel process (at optical wavelength of 635 nm [18]) and by laser ablation (at optical wavelengths from 400 nm to 900 nm, for *x* = 0, 0.3, 0.5 and 0.7 [19]). The sound velocity dependence on the composition, v=v(x), has been measured only in non-graded polycrystalline ceramics samples [20]. Both n=n(x) and v=v(x) are unknown for bulk single crystals of BSTx. Here we report the first measured dependencies on x of the optical refractive index n and of the sound velocity v in epitaxial BSTx films. The high-throughput characterization of the optical and acoustical properties is achieved via application for PLU of a custom asynchronous optical sampling (ASOPS) ultrafast lasers system [21]. We anticipate that PLU can be a rapid and nondestructive characterization technique for analyzing compositionally graded films.

### 1.2. Picosecond Laser Ultrasonics

Picosecond laser ultrasonics (PLU) is a branch of laser ultrasonics, where femtosecond or picosecond lasers are applied both for the generation and the detection of the coherent acoustic pulses (CAPs) [12,13,14,22,23,24]. For the conversion of the pump laser radiation into CAPs, its absorption in metals or semiconductors is commonly applied. The interband light absorption induces the mechanical stresses via electron-phonon deformation potential mechanism, while the transfer of the light energy absorbed by charged carriers to thermal phonons initiates thermo-elastic stresses [12,13,22,23,25]. These photo-induced mechanical stresses generate the CAPs of the nanoscale length if the pump light absorption region is of the nanoscale length. This condition is naturally satisfied by the skin depth of visible light in metals or its penetration depth in the semiconductors, if the pump optical quanta sufficiently exceed the optical band gap. Otherwise, for the spatial confinement of the pump light, its absorption in nanoscale-thick films or coatings can be used. The detection of the photo-generated CAPs is achieved by measuring the transient reflectivity of the probe laser pulses delayed in time relative to the pump laser pulses [12,22]. The reflectivity signals contain information on the CAPs because, on the one-hand, the probe light is partially scattered by the acoustic strain field via the acousto-optic effect [26] and, on the other hand, a CAP, propagating in the transparent layers can modify the thickness of the layers and thus can influence the reflectance of probe light via the interference effect [27]. Because the penetration depths of light in metals and semiconductors can be as short as 10 nm, the picosecond ultrasonics is a well-established technique for the evaluation of some parameters of the nanometers-thick films and coatings [28]. In particular, revealing the time of the CAP propagation across the film (echo method) [29] or the frequency of the film/coating mechanical vibrations [30,31] provides information on the ratio of the thickness to sound velocity. Revealing the acoustically induced interferometric modifications of the reflectivity provides information on the optical thickness of the film/coatings, i.e., the product of the thickness and the refractive index [32,33]. Additionally, a nano-CAP, being a moving reflector of probe light, modifies the frequency of the scattered probe light via the Doppler effect [34] or, saying this differently, because of the momentum and energy conservation laws in photon-phonon acousto-optic interaction [35]. The frequency shift of the scattered probe light, commonly called Brillouin frequency (BF), can be revealed in the transient reflectivity signal, when the CAPs are propagating in transparent media [12,13,14,23,24,36,37,38]. The transient reflectivity signal contains a component that is oscillating in time at the BF. The measurement of the BF provides information on the product of optical refractive index and sound velocity. The amplitude of the so-called Brillouin oscillations (BO) in the transient reflectivity is proportional to the acousto-optic (photo-elastic) constant. Thus, PLU provides access to optical, acoustical and acousto-optical parameters of nanometric films and coatings and can be profitably combined with other techniques of their characterization to extract the full set of required parameters.

### 1.3. Multi-Technique Approaches including Picosecond Laser Ultrasonics for Thin Film Characterization

PLU provides access to characterization of particular acoustical (sound velocity, v), optical (refractive index, *n*), acousto-optical (photo-elastic constant, *p*) and geometrical (thickness, *d*) parameters of thin films and coatings, which are influencing the transient reflectivity signals via their combinations *d*/v, dn and *n*v. Thus, the preliminary knowledge or independent measurement of *d*, *n* or v is required for the disentangling of these three parameters in PLU, unless the experiments are conducted at least at two different angles of probe light incidence [39,40]. However, commonly PLU experiments are conducted at a single (normal to the surface) incidence direction of probe light and the thickness of the films coatings is known from the calibration of their growth/deposition process. This is often not the case when the coatings of variable chemical/structural composition are under investigation. For example, for the experimental assessment by PLU of the elastic properties of a series of ternary transition metal nitride coatings M1_1−x_M2_x_N films (M1, M2 = Ti, Zr, Ta) [41], film thickness was preliminarily measured by X-ray reflectivity. The structural (texture and microstructure) and growth morphology evolution as a function of film thickness and chemical composition were investigated by X-ray diffraction, electron probe microanalysis and scanning electron microscopy. In the studies of thin films of the room temperature multiferroic Bi_1−*x*−*y*_Dy*_x_*La*_y_*FeO_3_ [42], the elastic moduli of rhombohedral, tetragonal, and rare-earth doped BiFeO_3_ were determined by PLU in conjunction with spectroscopic ellipsometry. In the recent experiments, for evaluating the effect of Praseodymium (Pr) substitution on the elasticity of multiferroic (Bi_1−x_Pr_x_)(Fe_0.95_Mn_0.05_)O_3_ (BPFMO) thin films [43], PLU was combined with optical spectral reflectometry, scanning electron microscopy and atomic force microscopy.

## 2. Methods and Experimental Results of Characterizing Laterally Graded Ba_1−x_Sr_x_TiO_3_ (BSTx) Library

### 2.1. Characterization of the Laterally-Graded Ba_1−x_Sr_x_TiO_3_ (BSTx) Library by X-ray Diffraction, Energy Dispersive Spectroscopy, Electron Probe Microanalysis and Scanning Electron Microscopy

**Figure 1:**
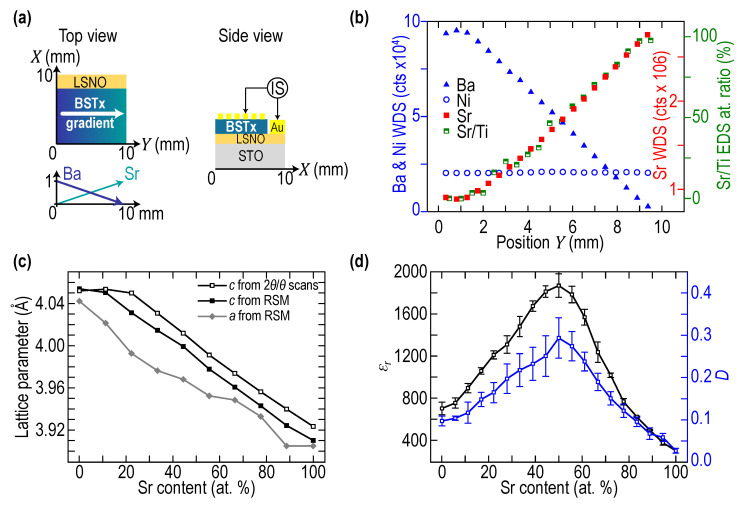
(**a**) Schematic of the BSTx laterally graded material library (top view) and of the heterostructure with top and bottom Au pads for impedance spectroscopy (side view). (**b**) EPMA WDS signal from Ba(Lα), Sr(Lα) and Ni(Kα) emissions as a function of position Y. Quantitative EDS Sr/Ti atomic ratio has been added for comparison (from Ref. [5] with permission). (**c**) In-plane and out-of-plane lattice-parameter dependence of BSTx on Sr concentration x and (**d**) dielectric permittivity ε_r_ and losses *D* versus x. Figure 1c,d are adapted with permission from Ref. [5]. Copyright 2016 Elsevier.

A schematic of the laterally graded Ba_1−x_Sr_x_TiO_3_ (BSTx) library is represented in Figure 1a. The gradient extends on 10 mm along the *Y* direction while the composition is constant along *X* direction. The BSTx film (250 nm nominal thickness) was grown on top of a homogenous conductive La_0.9_Sr_1.1_NiO_4_ (LSNO) layer deposited onto a (001)-oriented SrTiO_3_ single crystal. Detailed growth conditions are described in reference [5]. The growth of both layers was epitaxial, as it could be seen from X-ray diffraction (XRD) Reciprocal Space Maps (RSM) around (103)_STO_ reflection (see Figure 3 in reference [5]). The lattice mismatch between BSTx and STO substrate increases with Ba content, leading to strain relaxation in BSTx. The evolution of the BSTx in-plane *a* and out-of-plane *c* cell parameters, extracted from RSM and symmetrical Θ–2Θ XRD experiments [5], are reported in Figure 1b. Characterization by energy dispersive spectroscopy (EDS) of the Sr/Ti atomic ratio along the composition gradient was in good agreement with nominal composition [5]. It is however difficult to dissociate Ba and Ti overlapping peaks using an energy dispersive set-up, so we turned to electron probe microanalysis (EPMA) to further investigate the composition gradient. Indeed, Ba and Ti peaks were well separated in wavelength-dispersive spectroscopy (WDS). Qualitative WDS measurements along the gradient are reported for Ba, Sr and Ni in Figure 1c, together with the quantitative Sr/Ti atomic ratio from EDS from reference [5] for comparison purposes. The anti-correlated linear variations of Ba and Sr signals agree remarkably well with nominal composition variation, with a strong correlation between WDS Sr Lα peak intensity and EDS Sr/Ti atomic ratio. Furthermore, the constant signal of Ni, dependent on both the LSNO thickness (photon emission) and BSTx thickness (photon absorption) shows that both LSNO and BSTx are relatively homogeneous in thickness. The local thickness was evaluated by producing and observing cross-sections with focused ion beam and scanning electron microscopy, respectively, at several locations on the gradient. For the future convenience of the readers, the results of the thickness measurements are presented together with the laterally variable physical parameters of the film revealed by PLU in Section 2.2.3 (Figure 6c). All measured thicknesses belong to the 250 nm ± 10% interval. The targeted film thickness was 250 nm from the evaluation of BaTiO_3_ and SrTiO_3_ single target deposition rates using X-ray reflectometry. An array of 180 (9 × 20) Au top electrodes (180 × 500 μm^2^) was deposited through a shadow metal mask (Figure 1a) to locally measure the permittivity and dielectric losses by impedance spectroscopy (20 compositions measured 9 times) with an automated probe-station. The evolution of the permittivity ε_r_ and dielectric loss *D* versus Sr content x in BSTx is represented in Figure 1d. The maximum ε_r_ is found for x ≈ 50% which corresponds to the composition of a ferroelectric Curie temperature close to room temperature in bulk polycrystalline BSTx [6].

### 2.2. Characterization of the Laterally Graded Ba_1−x_Sr_x_TiO_3_ (BSTx) Library by Picosecond Laser Ultrasonics

Although several different strategies could be potentially used to determine the parameters of the transparent films of known thickness from the PLU signals, we found the acoustic velocities by measuring the times of CAPs propagation across the film and the refractive index from the measured Brillouin frequencies. Thus, the earlier unknown acoustical and optical parameters of the BSTO films in the complete range of x from our material library were determined. Optical parameters were determined at two different optical wavelengths of 356 nm and 535 nm applied in our experiments. From the physics point of view, our PLU experiments confirmed the expectations that the acoustical and optical parameters of the grown thin films could appreciably differ from those in the bulk materials of the same composition.

#### 2.2.1. Two-Laser Experimental Setup Based on Asynchronous Optical Sampling

**Figure 2:**
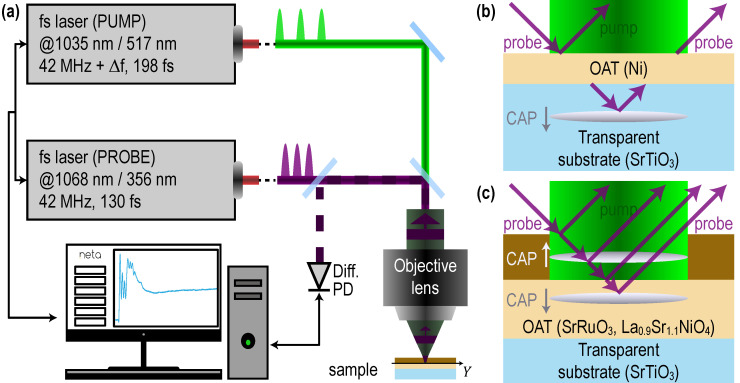
(**a**) Schematic presentation of a picosecond acoustic microscope based on asynchronous optical sampling. (**b**,**c**) Schemes of the optical rays and of the coherent acoustic pulses (CAPs) propagation in typical multilayered samples. OAT denotes pump light absorbing optoacoustic transducer, which launches CAPs due to an optoacoustic conversion initiated by the absorption of pump laser radiation. The top brown layer in (**c**) denotes the transparent epitaxial films of either the laterally homogeneous Ba_0.6_Sr_0.4_TiO_3_ material or the laterally graded Ba_1−x_Sr_x_TiO_3_ library material. Note that in parts (**b**,**c**), the probe beams are shown as oblique for a clearer representation of the reflections, while the probe beam was actually normal to the surface of the OAT.

The experimental setup is a commercial picosecond acoustic microscope (JAX-M1, NETA, Pessac, France) [21,44] based on asynchronous optical sampling (ASOPS). The pump and probe laser beams follow the same path and are co-focused on a part of the sample which absorbs light at the pump wavelength, called the opto-acoustic transducer (OAT), as shown schematically in Figure 2. Two pulsed fiber lasers of fundamental optical wavelengths of 1034.8 nm and 1068.4 nm, with pulse durations of 198 fs and 130 fs, respectively, and with a repetition rate of about 42 MHz are synchronized for the ASOPS [45]. The repetition rate of the follower laser cavity is slightly offset compared to that of the leader one. An offset of 500 Hz is used in our measurements, which corresponds to a temporal sampling of 0.28 ps. The fundamental radiations are then doubled and tripled in frequency, leading to the possibility of having 517 or 345 nm (from the follower cavity fundamental radiation) to use for the pump beam and 535 or 356 nm (from the leader cavity fundamental radiation) to use for the probe beam. Note that in Figure 2b,c, the probe beams are presented as obliquely incident for the convenience of the schematics, while both pump and probe beams were actually normally incident and co-focused on the surface of the OAT. The spot size at the focus was of approximately 1.25 µm radius at the 1/e^2^ level of the laser intensity when focused with a 50× objective lens or of 5 µm radius when focused with a 10× objective lens. The JAX-M1 picosecond acoustic microscope offers the possibilities of scanning the pump beam relative to the probe beam in the focal plane of the objective lens, which was used to assure a perfect overlap between both beams in our case. The sample was also mounted on a X-Y positioning stage equipped with step motors, ensuring the positioning precision of 0.16 µm which allows precise positioning of the tested sample and automatic scan along a line. Thanks to the ASOPS that automatically offset the arrival time of the probe pulse at the OAT compared to the arrival time of the pump pulse by a multiple of 0.28 ps, one measurement is accomplished within 2 ms. This is the time needed for the pump pulses (arriving every 23.80924 ns) and the probe pulses (arriving every 23.80952 ns) to be in phase again at the OAT surface. Because of this fast acquisition, there is no need of using a lock-in amplifier, as is usually done in pump-probe experiments using a mechanical delay line, and the signal-to-noise ratio here is simply improved by averaging 100,000 times (3 min and 20 s).

Since the ASOPS-based PLU measurement indeed took 3 min and 20 s per point, a total of about 2 h and 5 min of acquisition time was necessary to collect the complete set of data used in this study. Compared to the acquisition time nowadays achievable in the frequency-domain Brillouin microscopy done with a spectrometer based on virtual image-phased array (VIPA) technology, which is of less than or about 1 s to get one spectrum [46,47,48], the acquisition time reported here might seem large. Yet, this striking difference should be minimized for several reasons. First, in the case of samples made of a sub-micrometer-thin transparent film on a substrate, because the thickness of the film is less than the actual axial length of the probed volume in Brillouin microscopy, the scattered signal will diminish drastically, implying the need for integrating it over a longer time for good signal-to-noise ratio. Note that even if the use of the latest VIPA-based spectrometers or the stimulated Brillouin scattering technique could be faster, we were unable to find in the literature any application of those faster technologies with transparent thin films. Second, it should be mentioned that while we chose here to average our signals 100,000 times to get the best signal-to-noise ratio for our demonstration, this could be drastically diminished by a combination of the following possibilities: increase of pump and probe laser powers below the damage threshold, change of pump and probe laser wavelengths to improve the generation and detection efficiencies, increase of pump and probe pulse durations up to 10 times to minimize the peak power (hence allowing for higher powers) and to increase the spectral amplitude of the laser pulse intensity envelope at the frequencies of interest here (few tens of GHz), and/or change the repetition and the beating frequency of the ASOPS-based laser system. That being said, averaging over 10,000 acquisitions is a more typical value in ASOPS-based PLU leading to a measurement time of 20 s, and this is without changing any of the lasers parameters used. Finally, the PLU technique is a unique technique providing opportunity to determine simultaneously both optical and acoustical parameters (and, potentially, acousto-optic/photo-elastic parameters) of the films. The interferometry and ellipsometry obviously do not provide access to acoustic parameters, while frequency-domain Brillouin scattering (FDBS) measures the Brillouin frequency but is unable to disentangle the refractive index and sound velocity at a single angle of probe light incidence (as it is in our reported experiments) even if the information on the film thickness is available from preliminary measurements (for example, from the FIB-supported AFM measurements, as in our experiments). To get information equivalent to that accessible by PLU, FDBS should be combined with another technique, e.g., with the measurements of the film refractive index by interferometry/ellipsometry.

In the following sections presenting the results, three samples are tested with different experimental configurations. For the experiments on the bulk STO substrate, a 50× objective lens was used for three configurations: (i) 517 nm pump at 2 mW and 535 nm probe at 9 mW, (ii) 517 nm pump at 2 mW and 356 nm probe at 2 mW, (iii) 345 nm pump at 2 mW and 356 nm probe at 2 mW. For the experiments on the laterally homogeneous Ba_0.6_Sr_0.4_TiO_3_ thin film, a 50× objective lens was also used for the following configuration: (ii) 517 nm pump at 1 mW and 356 nm probe at 3 mW. Finally, for the experiments on the BSTx library, a 10× objective lens was used for two configurations: (i) 517 nm pump at 15 mW and 356 nm probe at 1 mW, (ii) 517 nm pump at 15 mW and 535 nm probe at 15 mW.

#### 2.2.2. Preliminary Experiments on Bulk STO and Homogeneous Ba_0.6_Sr_0.4_TiO_3_ Thin Film

Preliminary PLU experiments were conducted on bulk STO substrate and laterally homogeneous Ba_0.6_Sr_0.4_TiO_3_ thin film for further comparison with the results of the experiments on the laterally graded Ba_1−x_Sr_x_TiO_3_ (BSTx) library (Section 2.2.3). We present here these results for the convenience of the readers to illustrate the typical features of the PLU transient optical reflectivity signals in the case of laterally homogeneous substrates and films.

For the experiments in the transparent SrTiO_3_ (STO) substrate, Ni optoacoustic transducers of h≈ 10 nm thickness, i.e., thinner than pump light penetration length in bulk Ni, were deposited (Figure 2b). Ni was chosen because its acoustic impedance for the longitudinal sound waves is very close to the impedance of the STO substrate [49]. Thus, the bouncing of the photo-generated coherent acoustic pulses (CAPs) inside the transducer and corresponding contribution to the transient reflectivity signal at the thickness-resonance frequency of the transducer were reduced to minimum. The reflection of the CAPs photo-generated in Ni at the interface with STO did not exceed 14% [49]. Therefore, most of the coherent acoustic energy was transmitted into STO in a time of a single round trip of the longitudinal acoustic wave inside a nearly homogeneously and instantaneously laser-heated transducer ($2h/v_{Ni}\approx 3.3$ ps, for the velocity $v_{Ni}\approx 6$ nm/ps of the longitudinal acoustic waves in Ni). After this time, the acoustic contributions to the transient reflectivity signal were predominantly due to the CAP of about $2hv_{STO}/v_{Ni}\approx 27$ nm-length propagating in the STO (for the velocity $v_{STO}\approx 8$ nm/ps of the longitudinal waves in STO [50,51,52]).

**Figure 3:**
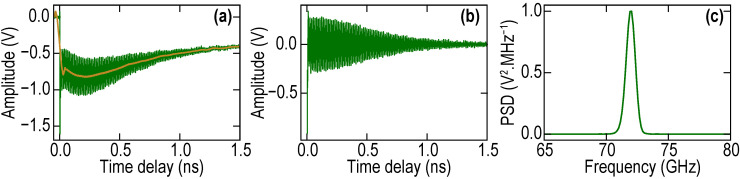
(**a**) Transient reflectivity signal, which follows Ni excitation by the pump pulse in the experiments conducted with pump wavelength of *λ* = 517 nm (green pump) and probe wavelength of *λ* = 535 nm (green probe). Number of averages is 10,000. (**b**) Acoustically induced transient optical reflectivity signal and (**c**) its power spectral density (PSD) evaluated in the complete temporal window (0–2 ns) of the experiments.

The detected signal shown in Figure 3a is delivered by the photodetector. It is proportional to the transient reflectivity signal ∆R/R to which we will refer in the following section when discussing the measured signals. The slowly varying background (indicated by orange line in Figure 3a) can be deleted by using a Savitzky-Golay filter with a window length of 351 points and third-order polynomial. This gives access to a part of the transient reflectivity signal related to acoustic waves in STO (Figure 3b) detected by the delayed probe laser pulse. The Fourier transform of the acoustically induced ∆R/R in Figure 3c clearly demonstrates the dominance of the Brillouin oscillation which is due to the probe light scattered by the acoustic pulse, which propagates in the STO substrate.

**Figure 4:**
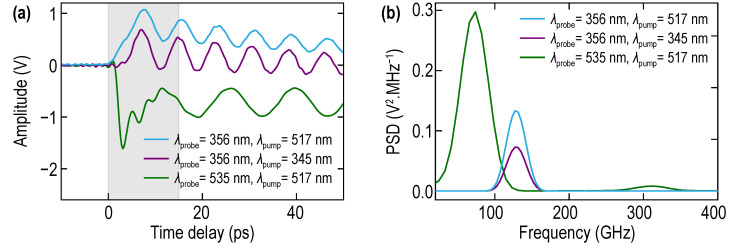
(**a**) Behavior of acoustic signal in the time window up to 50 ps after the sample photoexcitation for the different wavelengths of the pump and probe laser pulses and (**b**) its power spectral density (PSD) evaluated in the window from 0 to 20 ps, demonstrating the presence of a rather weak and damped vibration of the Ni layer at around 315 GHz detected by 535 nm probe light, corresponding to the half-wavelength acoustic resonance of the layer. The transient reflectivity signals and their spectra demonstrate the independence of the Brillouin oscillations frequency on the pump laser wavelength and its variations with the probe laser wavelength.

We searched for the signatures of the possible vibrations of the Ni layer, as acoustic impedance matching of the Ni layer to the substrate is not perfect. The data processing can be accomplished on the ∆R/R signal over a strongly reduced time interval after the pump laser pulse arrival (Figure 4a), where the signatures of the Ni film ringing could be expected. In this way, in the Fourier spectrum of the acoustically induced transient reflectivity signal, it is possible to reveal, when probing with green laser pulses, a peak at about 315 GHz frequency corresponding to the thickness resonance of the Ni optoacoustic transducer (see the right green spectral peak in Figure 4b). However, the magnitude of this peak is nearly an order of magnitude smaller than that at the Brillouin frequency (BF) (left green spectral peak in Figure 4b), as expected due to the small difference in the acoustic impedances of Ni and STO ($N_{Ni}/N_{STO}\approx 1.3$ [49]).

When we shortened the wavelength of the probe laser radiation down to λ = 356 nm (UV probe), the detected Brillouin frequency increased up to 128 GHz (blue and violet curves in Figure 4). The Brillouin oscillation frequency with UV probe is nearly twice as high as the one obtained with the green probe, confirming that we are able to monitor shorter acoustic wavelengths (down to 50 nm) in the bulk sample. This figure also provides an opportunity to compare the Brillouin frequencies in STO obtained with the blue and green pump, revealing that the BF does not depend on the wavelength of the pump laser as expected. Figure 4 additionally demonstrates that the vibrations of the Ni film are nearly invisible for UV probe (presumably because of the lower photoelastic constant of Ni for UV light in comparison with green (535 nm) light), providing an opportunity to test only the substrate even in the positions closest to the Ni film. In Figure 4, we shaded in gray the time interval where the influence of Ni oscillations on ∆R/R is visible in the case of the green probe light. In the case of the UV probe light (blue and violet curves in Figure 4a), this interval is more than three times shorter.

The frequency $f_{B}$ of the Brillouin oscillations (Figure 3 and Figure 4) is proportional to the product of acoustic velocity in the medium v and of the refractive index n of the medium at the probe wavelength λ [12,32,36,37]:

*f_B_* = (2*nv*)/*λ*. (1)

We used the dependence in Equation (1) for the estimates, providing confidence in the obtained results. The refractive index in STO at green wavelengths is well documented, $n_{STO}\left(535\;\mathrm{nm}\right)\approx 2.44$ [51,52,53,54]. Therefore, from the 72 GHz BF (Figure 3 and Figure 4), we extract v≈8 nm/ps. This velocity value is reliable for the STO substrate as it can be extracted also from the earlier PLU experiments conducted at different probe wavelengths [51]. We can estimate from the 128 GHz BF (Figure 4b) that the refractive index $n_{STO}\left(356\;\mathrm{nm}\right)\approx 2.91$. This value is consistent with the one quite recently measured by spectroscopic ellipsometry [54,55]. These preliminary experiments are important for the future comparison of the acoustic and optical properties of bulk STO with those from the STO in the laterally graded Ba_1−x_Sr_x_TiO_3_ (BSTx) library, i.e., at x = 0 composition point. They also provide an opportunity for straightforward identification in the spectra of the transient reflectivity signals of the contributions due to the STO substrate.

**Figure 5:**
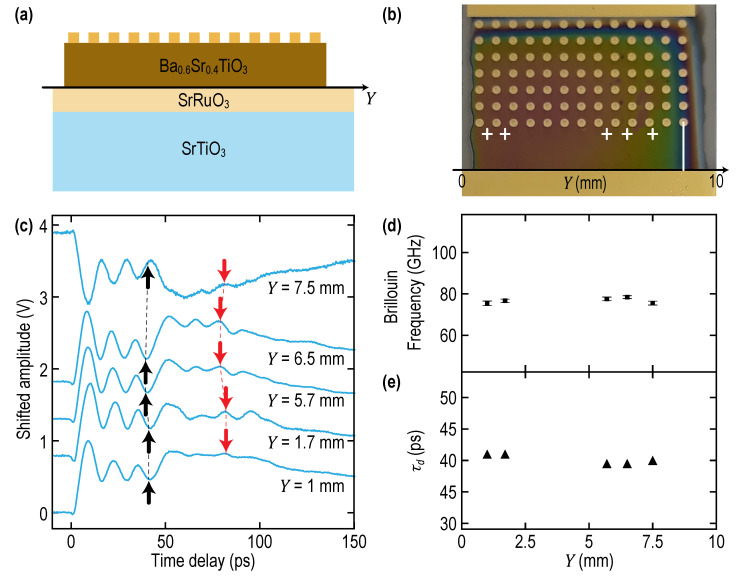
(**a**) The side view of the sample with BST0.4 thin film and (**b**) its optical image from the top of the sample, where the experimental points are marked by crosses. The variations in colors reflect the changes in the film thickness of less than 10–15% in the positions from 1 mm to 7.5 mm. (**c**) Transient reflectivity signals in the different lateral experimental positions. The Brillouin oscillations in the BST0.4 film are clearly visible. The arrivals of the CAP on the free surface of the film and its return to the SRO OAT are marked tentatively by black and red arrows (linked by dashed lines of the corresponding colors), respectively. (**d**,**e**) The dependences of the evaluated BF in BST0.4 and the estimated characteristic times $\tau_{d}\approx d/v_{BST0.4}$ of CAP propagation across the film, respectively, on the coordinate along the sample surface.

The second type of preliminary experiments were conducted on the Ba_0.6_Sr_0.4_TiO_3_ (BST0.4) thin films of 230 nm nominal thickness *d* obtained by epitaxial growth on top of SrRuO_3_ (SRO) optoacoustic transducer (60 nm-thick) deposited on the STO substrate. The cross-sectional view of the sample is similar to the one presented in Figure 1a but is presented for convenience in Figure 5a. The optical image obtained from the top side of the sample by smartphone is presented in Figure 5b, where the experimental points are indicated by crosses. The film is transparent for both pump and probe laser radiations, while the SRO transducer is opaque [56,57,58]. Thus, the experiment is conducted in the configuration depictured in Figure 2c.

In Figure 5c, the transient reflectivity signals obtained with the green pump and UV probe are presented, demonstrating a time behavior which is rather typical for PLU experiments with the transparent films [12,32,33,37,59,60,61]. The most important features of the signals in Figure 5c are:

1. The Brillouin oscillation is clearly visible in the first 30–40 ps and is due to the scattering of probe light by CAP propagating from the SRO optoacoustic transducer towards the free surface of the film (see Figure 2c). The frequency of this oscillation (about 77 GHz, corresponding to BF of the epitaxial BST0.4 film 40% lower than in STO substrate) is straightforward and distinguishable from the BF of 128 GHz in the STO substrate (Figure 4b). The latter could be revealed only as a small feature by the Fourier transform in the complete experimental time window (not shown). The experimentally revealed Brillouin frequencies are presented in Figure 5d. They were evaluated using the following fitting procedure. First, the raw signal is filtered from 7 ps to its end using a Savitzky-Golay filter with a window made of 121 points (~28.8 ps duration) and a polynomial of third order. Second, the filtered signal is subtracted to the raw one in order to only keep the acoustic contribution to the relative reflectivity changes, and a Fourier transform is done on this acoustic signal over a time interval spanning from 7 to 40 ps in order to obtain a first estimate of the Brillouin frequency. Third, this estimated frequency is used as a starting point for fitting the raw signal on the same time interval using the Levenberg-Marquardt algorithm to perform least squares minimization with a model signal of the form $Ae^{-\alpha t}\cos\left(2\pi f_{B}t+\varphi \right)+Bt+C$. Here the decaying exponential with parameter α accounts for the amplitude decay with time of the oscillations, φ is a phase shift and the affine function Bt+C allows to account for potential DC bias and slow overall amplitude variations owing to the non-subtracted slowly varying background. The revealed BF varies less than 4% relative to the average across the 7.5 mm distance along the BST0.4 film (Figure 5d). These results demonstrate the absence of significant gradients in BF and consequently in the film composition in the part of the sample with an almost homogeneous thickness, as could have been expected from the sample preparation.
2. The diminished amplitude of the BO in BST0.4, starting approximately after a 40 ps time delay, is a signature of CAP attenuation in propagation and in its reflection from the mechanically free rough surface [62,63].
3. The background signal, on which Brillouin oscillations are superimposed, exhibits a step-like variation in the process of CAP reflection from the free surface. This variation accompanies the variation of the BST0.4 film optical thickness. It modifies the so-called interferometric contribution of the CAP in the transient reflectivity signal. The signal features corresponding to the arrival time of the CAP on the mechanically free surface of the film $\tau_{d}\approx d/v_{BST0.4}$, when the strong variations in the background signals starts, are tentatively marked by black arrows in Figure 5c. Here, *d* denotes the transparent film thickness. The estimated arrival times are presented in Figure 5e. The arrival times and, consequently, the film thicknesses vary less than 3% relative to the average across the 7.5 mm distance along the BST0.4 film.
4. At time delays $2\tau_{d}$, which are about twice the time of the CAP reflection from the free surface and are tentatively marked by red arrows in Figure 5c, there are features in the signal due to the CAP incidence on the surface of the SRO transducer, which are both of photoelastic and interferometric origins. For example, the peaks in the transient reflectivity signal, following the time of CAP return to the SRO optoacoustic transducer, are commonly attributed to the photoelastic detection of the CAP transmitted inside the opaque transducer [60,61]. The photoelastic contribution to ∆R/R from the CAP reflected from the transducer could be significantly smaller because the photoelastic effect in a material, which is opaque for the probe light, is commonly stronger than in a transparent material. In particular, the stronger photoelasticity of the SRO for the probe light was reported in comparison with the rather common values of the photoelastic coefficients of the STO [59]. In our samples, the contribution to ∆R/R from the CAP reflected by SRO transducer is expected to be nearly completely suppressed due to the well-known close-to-perfect matching of the longitudinal acoustic impedance of SRO to that of STO and BaTiO_3_ (BTO) [46,49,64]. This expected, nearly perfect matching of the BST0.4 film, of the SRO optoacoustic generator and of the STO substrate is the reason for the absence of any pronounced temporal oscillations corresponding to the vibrational eigen modes of the film and of the optoacoustic transducer in the signals presented in Figure 5c [28,30,31,65,66].

The above discussion of the main features of the ∆R/R follows from the well-known theoretical formulas [12,14,23]. The optoacoustic transducer in the considered sample is thicker than probe light penetration depth in SRO [56]. Because of this, as long as we are not interested in the CAP propagation in the homogeneous STO substrate, the ∆R/R signal can be evaluated for the BST0.4 film on the SRO semi-infinite substrate [23]:

(2) $$\frac{\Delta R}{R}=4Im\langle \frac{r_{12}\left(1-r_{01}^{2}\right)}{\left(r_{01}e^{-ik_{1}d}+r_{12}e^{ik_{1}d}\right)\left(e^{-ik_{1}d}+r_{01}r_{12}e^{ik_{1}d}\right)}\{\left(k_{1}+\frac{\partial k_{1}}{\partial \eta }\right)\overset{d}{\underset{0}{\int }}\eta \left(Z,t\right)dZ+\;\frac{1}{2}\frac{\partial k_{1}}{\partial \eta }\overset{d}{\underset{0}{\int }}\eta \left(Z,t\right)\left[r_{12}e^{i2k_{1}\left(d-Z\right)}+\frac{1}{r_{12}}e^{-i2k_{1}\left(d-Z\right)}\right]dZ+\frac{1}{2}\frac{\partial k_{2}}{\partial \eta }\left(r_{12}-\frac{1}{r_{12}}\right)\overset{\infty }{\underset{d}{\int }}\eta \left(Z,t\right)e^{i2k_{2}\left(Z-d\right)}\}\rangle .$$

In Equation (2), the indices *i*, *j* equal to 0, 1 and 2 are used to denote the parameters of the air at *Z* < 0, the transparent film at 0 < Z < *d* and the optoacoustic transducer at *Z* > *d*, respectively. Thus, $r_{ij}$ is the reflection coefficient for the electric field of probe light, when the light is normally incident from the *i*-th to *j*-th part of the sample. $k_{i}$ is the probe light wave number in the *i*-th part. η(Z,t) is the acoustic strain field in the sample, which is evaluated by solving the problem of the CAP photo-generation by the pump laser pulses and the subsequent propagation of CAP inside the sample. The time independent coefficient in front of the figure bracket in Equation (2) accounts for the interference of probe light field inside the film. There are three different integral-type contributions inside the figure brackets in Equation (2). The first of them is proportional to acoustically induced variations in the transparent film thickness. It initiates time-dependent contributions to ∆R/R only when the CAP is interacting with the film surfaces. This term is responsible for the feature 3 of the signals in Figure 5c, when the CAP is reflected at the free surface at *Z* = 0. It also contributes to ∆R/R variations when the CAP is reflected/transmitted at the interface between the film and the substrate (contributes to feature 4) in Figure 5c. The second contribution inside the figure brackets is due to the photoelastic effect in the film. In the sufficiently thick film transparent for the probe light, it describes the Brillouin oscillation, i.e., the feature 1 of the signals presented in Figure 5c. In this case, the spatial Fourier factors $e^{\pm i2k_{1}Z}$ inside the integral of the second contribution select from the acoustic strain field η(Z,t) inside the film the acoustic waves with the wave vectors $2k_{1}$ (Brillouin wave vectors) corresponding to Brillouin frequencies of the acoustic waves in Equation (1). Finally, the third term in the figure brackets is due to the photoelastic effect in the substrate. In a substrate opaque for probe laser pulses, i.e., in the case of complex $k_{2}$ with the imaginary part comparable or exceeding its real part, this term could be most responsible for the pulse-like contribution to ∆R/R, i.e., the feature 4 of the signals in Figure 5c.

Using for the thickness of the BST0.4 its nominal value of 250 nm and roughly estimating from Figure 5d that the CAP crosses the film in 40 ps, the sound velocity in BST0.4 is estimated as $v_{BST0.4}\approx 6.25$ nm/ps. From Figure 5d, the BF in BST0.4 is around 77 GHz. Therefore, we estimate via Equation (1) the refractive index of the BST0.4 at our probe wavelength to be $n_{BST0.4}\left(356\;\mathrm{nm}\right)\approx 2.19$. Thus, in the BST0.4 sample, the sound velocity and the refractive index at the UV probe wavelength are both approximately 20% lower in comparison with the STO substrate. These estimates indicate that the parameters of the BSTx library could change appreciably, while PLU could be a sensitive tool for the characterization of the BSTx films with laterally varying composition (0≤x≤1).

#### 2.2.3. Characterization of the BSTx Library

**Figure 6:**
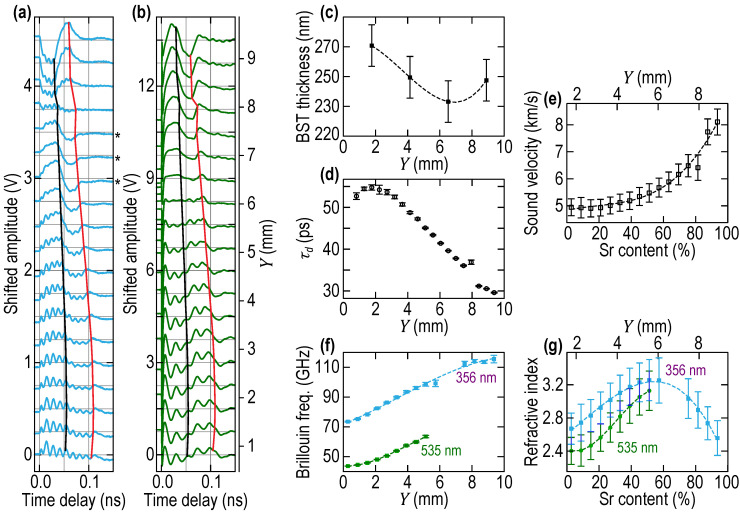
(**a**,**b**) Raw transient reflectivity signals detected at different lateral *Y* positions of the BSTx library using green pump and either (**a**) UV or (**b**) green probe. The black (red) solid lines connecting the raw signals vertically stand for the CAP arrival times $\tau_{d}$ at the free surface of the BSTx library layer (at the surface between the BSTx library layer and the OAT). (**c**) BSTx library thickness as a function of the position *Y* along the graded direction. (**d**) Time-of-flight $\tau_{d}$ of the acoustic wave propagating from the generator surface through the BSTx layer to its free surface estimated using the fitting procedure. (**e**) Sound velocity in the BSTx library as a function of the Sr content (bottom horizontal axis) and the position *Y* along the graded direction (top horizontal axis) estimated from the data in (**c**,**d**). (**f**) Brillouin frequencies as a function of the position *Y* along the graded direction of the BSTx library estimated using the fitting procedure for UV (blue experimental points) and green (green experimental points) probe. (**g**) Refractive index in the BSTx library as a function of the Sr content (bottom horizontal axis) and the position *Y* along the graded direction (top horizontal axis) estimated from the data in (**e**,**f**). The blue experimental points stand for the refractive index at the wavelength of the UV probe (356 nm), while the green ones are at the wavelength of the green probe (535 nm). Asterisks on the right of Figure 6a label the signals with the minimal interferometric and maximal photoelastic contributions, which are applied for the signal processing procedure suggested in Section 3.1.

The preparation of the BSTx laterally graded film and its characterization by the technique complementary to PLU are described in Section 2.1. The only result presented in Figure 6, which was obtained not with PLU but with FIB and SEM, is the thickness measurements presented in Figure 6c. The BSTx film thickness *d* depends on the position *Y* at the sample, i.e., it is different in the positions with different Sr content x. It is obtained by continuous third order polynomial approximation to the thickness measurements in four different points. It is presented here for the convenient comparison of the lateral variations in thickness of the BSTx film with the lateral variations of the parameters accessed by PLU, and for the appreciation of the fact that the uncertainty in the determination of the sound velocities and refractive indices by PLU is dominantly controlled by the uncertainty in thickness measurements. It is commonly considered that the positioning of the interface with SEM can be achieved with the uncertainty of δZ≈ 10 nm [43]. Relating the uncertainty in the film thickness to the uncertainty in positioning of its two surfaces by $\delta d=\sqrt{{\left(\delta Z\;\right)}^{2}+{\left(\delta Z\;\right)}^{2}}=\sqrt{2}\delta Z$, we estimated that depending on the *Y* coordinate, it varies between ±5% and ±6%.

The PLU experiments were conducted similarly to those on BST0.4 film described in Section 2.2.1 (Figure 2c). The sample dimensions were the same, while the side view and the optical image of the BSTx sample from the top were similar to the ones presented in Figure 5a,b, respectively. However, to characterize lateral inhomogeneity of the sample, the ∆R/R signals were accumulated in significantly larger number and closer spatial points along the line of the scan. In Figure 6a,b, the signals registered with, respectively, UV and green probe light are presented in 19 points separated by 0.4755 mm steps, starting from the coordinate *Y* = 0.8132 mm of the sample.

Comparison of the signals presented in Figure 6a,b with those in Figure 5c indicates that the BTSx library is laterally inhomogeneous, as expected from its preparation. Please note that the signals presented in Figure 6a,b are the raw signals of transient reflectivity, while the vertical and horizontal gray lines are just for guiding the eyes. The continuous variation with the lateral coordinate *Y* (shown as a vertical axis on the right of Figure 6b) of the CAP arrival times at the free surface and the return times to the OAT are indicated in these figures by black and red curves, respectively. Please compare their continuously varying positioning in the temporal profiles of the ∆R/R signals to the positioning of the black and red dashed curves in Figure 5c, carrying the same physical sense. The dashed curves to guide the eyes in Figure 5c are nearly parallel between themselves and also to the vertical axis for the same order of the distance between the first and the last signal, as in Figure 6a,b. The arrival times of the CAPs on the mechanically free surface of the BSTx library presented in Figure 6d diminish by more than 45% from the BTO side of the sample (bottom of Figure 6a,b) to the STO side of the sample (top of Figure 6a,b). These variations are nearly one order of magnitude larger than those observed in the BST0.4 of the same thickness. Please note that the arrival times in BSTx library were determined not by eyes guided by the theoretical expectations (as they were estimated in BST0.4), but through the application of the fitting of the dedicatedly developed theoretical formulae for the interferometric part of the ∆R/R in Equation (2) to process raw signals detected by UV probe. The motivations for applying a more formal approach to the evaluation of the CAP propagation time across the film are described in the Section 3.1, where the theoretical formulas developed for fitting are also presented together with some illustrations. Here we note that the $\tau_{d}$ determined through the fitting procedure differs from the $\tau_{d}$ determined by “educated” eyes by about 0.5 ps at the STO edge of the sample and about 2.5 ps at the BTO edge. The maximum difference of 4.5 ps was estimated in the experimental position 17 (close to *Y* = 8 mm coordinate), where the contribution of the Brillouin oscillations to ∆R/R dominates over the interferometric one (see Figure 6a), indicating that interferometric detection of film thickness variations by UV probe is close to its zero sensitivity point and, correspondingly, ∆R/R can be a nonlinear function of the film thickness variations. We believe that the fitting procedure close to *Y* = 8 mm coordinate is not efficient because theoretical formulae assume a linear dependence on the film thickness of the interferometric signal. Smoothing the kink in the black line of Figure 6a and in Figure 6d in the considered point provides an opportunity to estimate that fitted $\tau_{d}$ does not deviate from $\tau_{d}$ determined by eyes by more than 9% over the complete dimension of the BSTx library. Thus, in view of the above estimated uncertainty in the film thickness measurements, i.e., ± 5−6%, $\tau_{d}$ determined by educated eyes could be applied, if required, to the estimates of the sound velocity in the film, as it was done at the end of Section 2.2.2. Note that the 66% confidence intervals in the determination of $\tau_{d}$, reported as error bars in Figure 6d, were estimated from the standard deviation error of the fitting procedure, which is detailed in Section 3.1. As described in Section 3.1, significant roughness of the BSTx film surfaces modulates the propagation time of the acoustic rays across the film and smooths sharp features (kinks) in the interferometric contribution to ∆R/R(t), which could be in its absence used for much more certain measurement of the arrival times. The maximum uncertainty of ±1.1 ps in the determination of $\tau_{d}$ corresponds to the maximum uncertainty in the propagation distances of ±8.9 nm (estimated with the maximum sound velocity, $v_{STO}\approx 8.1$ nm/ps, in our films) is caused by surface roughness but is appreciably smaller than the root mean square (RMS) of the surface roughness measured with an AFM on the mechanically free surface of BSTx and evaluated simultaneously with $\tau_{d}$ in the fitting procedure.

In Figure 6e, we present the determined dependence of the longitudinal sound velocity on the composition parameter x, obtained by combining our spatially resolved measurements of $v_{BSTx}=d\left(x\right)/\tau_{d}\left(x\right)$ with the dependence of the composition on spatial coordinates presented in Figure 1b. The longitudinal sound velocity as a function of x is obtained with the previous equation from the 19 measured values of $\tau_{d}$ and the values of the thickness extrapolated from the four measurements. The uncertainty in the determination of sound velocity, controlled mostly by the uncertainty in thickness measurements, does not vary much with the increase of sound velocity in Figure 6e: from 6.3% at the BTO edge of the sample to 5.9% at the STO edge of the sample. In Figure 6f, we present the dependencies, revealed by PLU, of Brillouin frequencies in the BSTx library on the coordinate *Y* at two different optical probe wavelengths. The BFs were obtained using a similar procedure of signal processing as the one presented in Section 2.2.2. However, instead of fitting the raw signal, we used the fact that we fitted the interferometric contribution in ∆R/R (see Section 3.1), therefore the contribution can be subtracted from the raw signal. Then, the fit of the resulting background-free signal is accomplished with the least-squares-minimization approach (with the Levenberg-Marquardt algorithm) using $Ae^{-\alpha t}\cos\left(2\pi f_{B}t+\varphi \right)+C$ as a model signal. Note that in the case of the green probe, the background incorporates an extra linear-in-time variation which is accounted for in the fit of the background and therefore removed from the signal when the fit is done to extract the BF. Note that after the position *Y* of ~5.5 mm, the Brillouin oscillation contribution to the signal is too small to allow trustable fits, explaining why the BFs are not extracted for the green probe all along the BSTx library (see Figure 6f). The 66% confidence intervals in the BF, reported as error bars in Figure 6f, are estimated from the standard deviation error of the fit. Finally, in Figure 6g, the optical refractive indices in BSTx library are presented, as obtained from Equation (1) and the combination of Figure 6e,f, with the confidence intervals being obtained from the velocities and BFs.

## 3. Discussions

### 3.1. On the Role of the Surface Roughness

**Figure 7:**
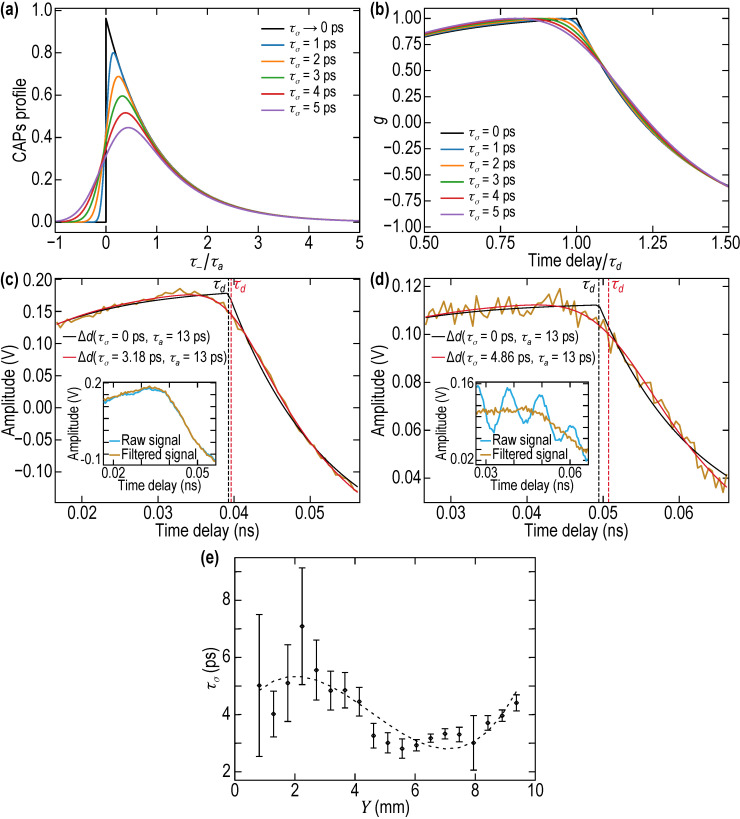
(**a**) Evolution of the CAPs profile associated with the increase of the surface roughness for $\tau_{\sigma }$ between 0 ps (no roughness) and 5 ps. (**b**) Evolution of the temporal profile *g* of the thickness variations near the smoothed kink at $t=\tau_{d}$ as a function of surface roughness for the values $\tau_{d}=40$ ps and $\tau_{a}=13$ ps. (**c**,**d**) Comparison of the filtered signals (orange) with the results of a fit with (red) and without (black) accounting for the surface roughness for two different types of signals detected with UV probe: (**c**) low and (**d**) large contribution of the Brillouin oscillations to the signal. (**e**) $\tau_{\sigma }$ as a function of *Y* position along the graded direction of the BSTx library.

In the leading approximation, the strain CAP generated by pump light absorption in opaque La_0.9_Sr_1.1_NiO_4_ (LSNO) media near its interface *Z* = *d* with the transparent BSTx film and launched into the film can be approximated by $\sim \theta \left(\frac{Z}{v_{BSTx}}+t-\tau_{d}\right)e^{-\frac{\frac{Z}{v_{BSTx}}+t-\tau_{d}}{\tau_{a}}}\equiv \theta \left(\tau_{-}\right)\exp\left(-\frac{\tau_{-}}{\tau_{a}}\right)$. Here θ denotes the step function, $\tau_{a}=l_{pi}/v_{LSNO}$ is the characteristic duration of the CAP and $l_{pi}$ is the characteristic spatial scale along *Z* coordinate (depth) of the stresses photo-induced by the absorption of the femtosecond pump laser pulse at time *t* = 0. This temporal profile is one of those presented in Figure 7a. The description of the strain field is valid in the transparent film 0≤Z≤d when $0\leq t\leq \tau_{d}\equiv d/v_{BSTx},$ i.e., before the arrival on the surface of the instantaneous leading front of the CAP. The reflection coefficient for strain at mechanically free surface is equal to −1. Therefore, in the time interval $\tau_{d}\leq t\leq 2\tau_{d}$, the strain in the transparent film 0≤Z≤d is described by [23] $\sim [\theta \left(Z+v_{BSTx}t-d\right)e^{-\left(Z+v_{BSTx}t-d\right)/\tau_{a}}-\theta \left(Z-v_{BSTx}t+d\right)e^{-\left(Z-v_{BSTx}t+d\right)/\tau_{a}}]$, where the second term in the square brackets accounts for the reflected pulse of the CAP, before its incidence on the OAT. The substitution of these strain profiles in the second term of Equation (2) provides the description of the interferometric contribution to ∆R/R in the time interval $0\leq t\leq 2\tau_{d}$, which is proportional to the variations of the film thickness:

(3) $$\Delta d\left(t,\tau_{a}\right)\sim d\left(\frac{\tau_{a}}{\tau_{d}}\right)\{\begin{array}{c} 1-e^{-\frac{t}{\tau_{a}}},\;when\;0\leq t\leq \tau_{d},\\ 2e^{-\frac{\left(t-\tau_{d}\right)}{\tau_{a}}}-1-e^{-\frac{t}{\tau_{a}}},when\;\tau_{d}\leq t\leq 2\tau_{d}. \end{array}$$

This temporal profile is one of those presented in Figure 7b. The solution in Equation (3) demonstrates that the dynamics of the interferometric contribution to ∆R/R are controlled by two characteristic times, $\tau_{a}\;$and $\tau_{d}$, and that there is a kink in the temporal behavior of Δd at $t=\tau_{d}$: $\partial \Delta d/\partial t\left(t=\tau_{d}-0\right)-\partial \Delta d/\partial t\left(t=\tau_{d}+0\right)\sim 2\neq 0.$ This abrupt change in the signal derivative is not observed experimentally (see Figure 6a,b and Figure 7c,d), indicating that the above theory does not take into account the phenomena that could lead to smoothing the revealed kink. These phenomena include the broadening of the leading front of the CAP caused by the CAP absorption in the propagation across the film. The broadening of the photo-generated CAP could be also caused by the roughness of the interface at *Z* = *d*, while the front of the CAP, reflected from the film surface at *Z* = 0, could be additionally broadened because of this free surface roughness.

The acoustic absorption in STO in the GHz frequency range is well documented [67]. It is quadratic in acoustic frequency. For the sake of the estimates, the experimental data on the mean free path of the acoustic phonon on frequency *f* (reported in Figure 7 of [67]), which is inverse of its absorption coefficient $\alpha_{a}\left(f\right)\sim f^{2}$, can be approximated as $\alpha_{a}^{-1}\left(f\right)≅10\;\mu m{\left(\frac{f}{100\;\mathrm{GHz}}\right)}^{-2}$ or $\alpha_{a}=a\omega^{2}$ with$\;a≅2.5\times 10^{-19}$ s^2^/m and ω denoting the cyclic frequency. Absorption, which is quadratic in frequency, transforms an instantaneous leading front of the photo-generated CAP $\sim \theta \left[\left(Z-d\right)/v_{BSTx}\right]$ at *t* = 0 into the front $\sim \mathrm{erf}\left[Z/(\sqrt{2ad}v_{BSTx}\right)]\theta [Z/v_{BSTx}]\;$at *t* = $\tau_{d}$, with the characteristic duration $\tau_{fa}\equiv \sqrt{2ad}$. Thus, in our experiments, this broadening of the CAP front by acoustic attenuation is $\tau_{fa}≅0.35\;\mathrm{ps}$. It is comparable with the duration of laser pulses but is much smaller than the characteristic time interval of the kink smoothing, observed in the experiment of ≈2.5 ps. Therefore, the broadening of the kink is expected to be caused by the roughness of the surface at *Z* = 0 and of the interface at *Z* = *d*. Potentially both could contribute because the nominal thicknesses of BSTx film (≈250 nm) and of LSNO layer (≈120 nm) are comparable. However, the roughness of the reflecting surface *Z* = 0 “plays” twice in comparison with the roughness of the transmitting surface *Z* = *d*, because the CAP is traversing the roughness of the free surface twice but the roughness of the OAT generator only once before the theoretical expected time interval of the appearance of the kink.

The roughness of the BSTx surface was characterized by AFM in three different positions on the film at the coordinates *Y*≈1, 4, 7 mm. The root mean square (rms) roughness was measured as $\sigma_{rms}\approx 11,\;13,\;15$ nm, respectively. These measured values agree with measurements of the rms roughness from 6 nm [68] to 25 nm [69] on the films of similar materials and of similar thicknesses, although deposited under different growth conditions. In [69], the roughness was measured on the BST0.2 films deposited on Pt, Si and Si/SRO at oxygen pressure of 3 Pa, which is significantly lower than the oxygen pressure used during the deposition of our BSTx library (10 Pa), while a rather strong dependence on the deposition pressure of the surface roughness is known [69,70]. In [69], (Ba,Sr)TiO_3_ films were deposited on MgO substrate, hence precluding direct comparison with our deposition on STO/LSNO. In addition, the rms surface roughness measured by AFM depends in general on the surface area of the film tested by AFM, on the distance between the sampling points, etc. [71], which vary from one reported experiment to another. In our measurements, the rms roughness in the same positions Y≈1, 4, 7 mm diminished approximately two to three times when the tested surface area diminished by an order of magnitude. Therefore, in the absence of the opportunity for an explicit comparison of our AFM measurements to earlier reported results, the possibility to estimate the surface roughness from the PLU experiment is an additional motivation for the evaluation of the surface roughness influence on the acoustic contribution to ∆R/R in PLU experiments.

The longitudinal sound velocities around the coordinates *Y* ≈1, 4, 7 mm are $v_{BSTx}\approx 5,\;5.2,\;6.2$ nm/ps, respectively. Thus, the characteristic time scales determined as $\tau_{rms}=\sigma_{rms}/v_{BSTx}$ can be estimated in these positions as $\tau_{rms}\approx$2.2, 2.5, 2.4 ps, respectively. These values are all of the same order as those determined by eye intervals of kink smoothing in the ∆R/R signals, indicating that the surface roughness is indeed a plausible explanation for the observed smoothing. However, to estimate the rms surface roughness from the PLU experiments and to evaluate the role of roughness more quantitatively to understand its influence on the determination of the characteristic propagation times of CAPs across the BSTx film thickness and on the uncertainty of this determination from the temporal positions of the smoothed kinks in the temporal ∆R/R signals, a more formal mathematical model is required.

We propose the simplest theoretical model, which treats the propagation between the opposite sides of the BSTx film of the transversely local parts of the CAP as the propagation of the rays normally to average interfaces with the phases chaotically modified by the surface roughness. In this model, each cyclic frequency component ω of the locally emitted-by-OAT CAP profile ($\sim \theta \left(\tau_{-}\right)\exp\left(-\frac{\tau_{-}}{\tau_{a}}\right)$) acquires an additional phase shift $\left(\frac{\omega }{v_{BSTx}}\right)\Delta Z$, where ΔZ is the chaotic variation of the film thickness caused by roughness of the surfaces. Assuming Gaussian (normal) distribution of ΔZ probability along the BSTx film with the standard deviation σ ($P\left(\Delta Z\right)=\frac{1}{\sqrt{2\pi }\sigma }\exp\left[-\frac{1}{2}{\left(\frac{\Delta Z}{\sigma }\right)}^{2}\right]$) the averaging of the factor $\exp\left[i\left(\frac{\omega }{v_{BSTx}}\right)\Delta Z\right]$ in the CAP spectrum reduces to the multiplication of each spectral component of the CAP by the Gaussian factor $\exp\left[-\frac{1}{2}{\left(\frac{\sigma }{v_{BSTx}}\omega \right)}^{2}\right]\equiv \exp\left[-\frac{1}{2}{\left(\tau_{\sigma }\omega \right)}^{2}\right]$, i.e., an effective quadratic frequency attenuation of the spectral components of the CAP. The temporal profile of the CAP, which, in the absence of the surface roughness, is an instantaneous front followed by an exponential decay, is transformed into $\sim \frac{1}{2}\exp\left[-\frac{1}{2}{\left(\frac{\tau_{\sigma }}{\tau_{a}}\right)}^{2}\right]\left\{1+\mathrm{erf}\left[\frac{1}{\sqrt{2}}\left(\frac{\tau_{-}}{\tau_{\sigma }}-\frac{\tau_{\sigma }}{\tau_{a}}\right)\right]\right\}\exp\left(-\frac{\tau_{-}}{\tau_{a}}\right)$. The evolution of the CAP profile with increasing ratio $\tau_{\sigma }/\tau_{a}$ is depictured in Figure 7a. The modification of the pulse profile leads to the modification of Equation (3) of the BSTx film thickness, to which the interferometric contribution to ∆R/R is proportional:

(4) $$\Delta d\left(\theta ,r,p\right)\sim g\left(\theta ,r,p\right)=\frac{d}{2r}\langle -2\mathrm{erf}\left(\frac{\theta }{\sqrt{2}p}\right)+\mathrm{erf}\left[\frac{1}{\sqrt{2}p}\left(\theta -r\right)\right]+\mathrm{erf}\left[\frac{1}{\sqrt{2}p}\left(\theta +r\right)\right]+e^{\frac{1}{2}p^{2}-\theta }\left\{2-e^{r}-e^{-r}+\;2\mathrm{erf}\left[\frac{1}{\sqrt{2}}\left(\frac{\theta }{p}-p\right)\right]-e^{r}\mathrm{erf}\left[\frac{1}{\sqrt{2}}\left(\frac{\theta }{p}-p-\frac{r}{p}\right)\right]-e^{-r}\mathrm{erf}\left[\frac{1}{\sqrt{2}}\left(\frac{\theta }{p}-p+\frac{r}{p}\right)\right]\right\}\rangle .$$

For compactness, we introduced in Equation (4) the normalized time shifted by $\tau_{d}$, $\theta =\frac{t-\tau_{d}}{\tau_{a}}$, and the dimensionless parameters $r=\frac{\tau_{d}}{\tau_{a}}$ and $p=\frac{\tau_{\sigma }}{\tau_{d}}=\frac{1}{r}\left(\frac{\tau_{\sigma }}{\tau_{a}}\right).$ In Figure 7b, we present how the temporal profile g of the thickness variations with the kink at $t=\tau_{d}$ (Equation (4)) is modified by the presence of the surface roughness with increasing ratio $\tau_{\sigma }/\tau_{a}$ for the typical values $\tau_{d}=40$ ps and $\tau_{a}=13$ ps revealed in our experiments with the BSTx library. The normalization of the time delay axis by $\tau_{d}$ allows to appreciate how the smoothing of the kink is shifting to a smaller time the maximum of the interferometric contribution compared to the case without any roughness. Hence, while visually estimating the time of CAPs arrival at the free surface, it is clear that we are underestimating its value.

The theoretical formula in Equation (4) was applied to fit the experimental profiles of ∆R/R after filtering out the oscillation at the Brillouin frequency $f_{B}$ with a second-order digital notch filter (numerator coefficients: $b_{0}=1$, $b_{1}=-2\cos\left(2\pi f_{B}/f_{s}\right)$, where $f_{s}$ stands for the sampling frequency, $b_{2}=1$; denominator coefficient: $a_{0}=1$, $a_{1}=-2\rho_{0}\cos\left(2\pi f_{B}/f_{s}\right)$, $a_{2}=\rho_{0}^{2}$, where $\rho_{0}$ has been set to 0.92) centered at the Brillouin frequency estimated with a Fourier transform (see the inset showing raw and filtered signals in Figure 7c,d). The results of the fit are presented as red curves for two types of the signal, with low and large contribution of Brillouin oscillations to the detected signals, measured with the UV probe in Figure 7c,d, respectively. Because of the differences in the raw data for different *Y* positions and for both probe wavelengths, we extracted the different characteristic times in Equation (4), namely $\tau_{a},\;\tau_{d}$ and $\tau_{\sigma }$, using the following fitting procedure in several steps. Because the background variations for the signals detected with the UV probe were much smaller than in the case of the green probe, the signals detected with the former were used for extracting the characteristic times.

First, assuming that the characteristic duration of the CAP ($\tau_{a}$) does not depend on *Y*, we selected the positions where the interferometric contribution to ∆R/R are maximal and other contributions (Brillouin oscillations and slowly varying background) are minimal: those selected positions, labeled by an asterisk on the right of Figure 6a, are at *Y* = 6.5192, 6.9947, 7.4702 mm. The three selected signals were fitted in the fitting region $\tau_{d}-20\;\mathrm{ps}\;\leq t\leq \tau_{d}+20\;\mathrm{ps}$ with the model signal AΔd(θ,r,p)+DC where *A* is an amplitude constant, and *DC* is a constant shift under the constraint that $\tau_{a}$ is equal for all. The fit was performed using the lmfit python library with a Levenberg-Marquardt algorithm to perform the minimization [72]. The fitting region was chosen to avoid the increasing background gradients at shorter times and the influence on signal of the CAP penetration in and reflection from OAT at larger times, which were not accounted for in Equation (4). The obtained value for $\tau_{a}$ is 13 ± 0.5 ps.

Second, in order to extract *τ_d_* and $\tau_{\sigma }$ as a function of *Y*, all signals (with UV probe), filtered with a notch filter centered on the estimated Brillouin frequency at each *Y* position, were fitted using the same model signal and minimization function as previously, but with a fixed value of $\tau_{a}=13$ ps. The extracted $\tau_{\sigma }$ are depicted in Figure 7e as a function of *Y*, while the values of $\tau_{d}$ are in Figure 6d. In Figure 7c,d, the comparison between the fitted curves without and with the roughness effect (black and red curves, respectively) stresses out the importance of accounting for the roughness in our fits to obtain a good estimate of the time-of-flight $\tau_{d}$. It can be seen that the value of $\tau_{d}$ increases when the roughness is taken into account, not mentioning the obviously better fit of the background in that case. The reported error bars in Figure 7e were obtained using the 66% confidence interval estimated from the standard deviation error of the fitting method. By comparing the error bar variations for $\tau_{d}$ in Figure 6d and that for $\tau_{\sigma }$ in Figure 7e, it is clear that they are correlated. This takes place because both parameters are fitted at the same time and therefore their confidence interval is a direct image of the goodness of the fit.

The characteristic time $\tau_{\sigma }$ determined from our PLU measurements (Figure 7e) in combination with the measurements of the sound velocity (Figure 6e) provides opportunity to characterize the roughness of the BSTx films and to compare this characterization with the one obtained by AFM. In accordance with the measurements, $\tau_{\sigma }\approx 4.5,\;4.6,\;3.3$ ps in the positions Y≈1, 4, 7 mm characterized by AFM. Multiplying by the sound velocities, we find the standard deviations σ≈22, 24, 20 nm. The rms surface roughness is $\sqrt{2/\pi }$ smaller, $\sigma_{rms}\approx 17,\;19,\;16$ nm. Therefore, the rms surface roughness estimated from the PLU experiments is just about 35% larger than measured by AFM ($\sigma_{rms}\approx 11,\;13,\;15$ nm, respectively), when averaged over the three experimental points. We consider this correlation to be a strong argument in favor of possible quantitative characterization of surface roughness of the films by PLU. It is tentative to diminish this difference between the two measurements by taking into account that the surface area probed by our Gaussian laser beams with the radii of 5 µm is π times larger than the surface area of 5 µm × 5 µm, evaluated by AFM. However, this would still not provide proof that PLU could measure the surface roughness precisely, because the rms evaluated by the AFM is the characteristic of the free surface of the BSTx film, while the rms determined by PLU is a combined characteristic of the two opposite surfaces of the BSTx film. In the absence of the measurements of the LSNO OAT roughness before the BSTx deposition, it is currently impossible to go to deeper comparisons. The only conclusion that could be done additionally is that the values of rms measured by PLU, close to those measured by AFM, is the indication that the roughness of the LSNO surface is either correlated to that of the free surface of the BSTx film rather than anti-correlated or is just small.

To conclude this discussion on the characterization of the surface roughness, it is worth mentioning that the precise correlation/comparison of the surface roughness characterization by two different measurement techniques is very difficult if ever possible. The characterization of the surface roughness by PLU could be important for the characterization of the individual films/layers which could be used in the design of other optoacoustic experiments, for example when composing multi-layered structures (growing the superlattices). In this case, PLU measurements of the roughness would provide access to the quantitative prediction of the CAPs propagation in these structures, while the measurements by AFM could support only qualitative predictions.

The PLU was already applied earlier for the characterization of the nanoscale surface with rms down to an order of magnitude smaller than in our samples (see [62,63] and the references therein). However, in these experiments, either the reflections of quasi-monochromatic coherent acoustic wave packets from the rough surface or the influence of the surface roughness on the quality factors of the acoustic eigen modes of the free-standing nano-membranes were monitored. Here we have accessed the characterization of the surface roughness with the wide-frequency-band CAPs at GHz frequencies.

### 3.2. On the Revealed Duration of the Photo-Generated Coherent Acoustic Pulse

Our fitting procedure revealed that the characteristic duration of the CAP launched into BSTx library from pump light absorbing La_0.9_Sr_1.1_NiO_4_ OAT is about $\tau_{a}^{exp}\approx \;$13 ps. This observation can be compared with the theoretical expectations that CAP duration is controlled by the time of sound propagation across the depth distribution of the photo-induced stresses, while the characteristic scale of the distribution is controlled by the pump light absorption depth $l_{OAT}^{green}$ in La_2−x_Sr_x_NiO_4_, $\tau_{a}^{theor}\sim l_{OAT}^{green}/v_{OAT}$, where $v_{OAT}$ denotes the sound velocity in our OAT. Although the physical parameters of La_2−x_Sr_x_NiO_4_ required for the quantitative theoretical estimate are very poorly known and those for the particular composition parameter x = 1.1 of our LSNO OAT are unknown, the qualitative discussion of our observation would be still insightful.

In [73], it is reported that the optical conductivity La_0.6_Sr_1.4_NiO_4_ at our pump wavelength of 517 nm (pump photon energy of 2.4 eV), $\sigma_{x=1.4}^{green}\approx 300\;{\left(Ω\mathrm{cm}\right)}^{-1}$ is practically independent of light polarization direction relative to the crystalline axis of the material. A rather weak dependence of optical conductivity on green light polarization was also reported in [74]. The measured optical conductivities increased from $\sigma_{x=0.3}^{green}\approx 300\;{\left(Ω\mathrm{cm}\right)}^{-1}$ to $\sigma_{x=0.5}^{green}\approx 450\;{\left(Ω\mathrm{cm}\right)}^{-1}$. Assuming that the optical conductivity of La_0.9_Sr_1.1_NiO_4_ for green light is the same order as those reported in [73,74], $\sigma_{x=1.1}^{green}\approx 450-300\;\left(Ω\mathrm{cm}\right){\;}^{-1}$, we estimated the pump light penetration depth in our OAT as $\left(l_{OAT}^{green}\right)\approx 70-90\;\mathrm{nm}$. We have not found any reports on the measurements of the sound velocity in La_2−x_Sr_x_NiO_4_. However, in our experiments, we did not observe any frequencies in acoustically induced ΔR/R that could be related to resonantly oscillating La_0.9_Sr_1.1_NiO_4_ and BSTx layers, either separately or together. We also did not observe any signatures of the return to the La_0.9_Sr_1.1_NiO_4_/BSTx interface of the CAP, initially launched in LSNO, after its partial reflection at the interface of the La_0.9_Sr_1.1_NiO_4_ with the substrate, i.e., with STO (se Figure 2c). These are the indications of the close matching between the acoustic impedances of the films and the substrate materials, similar to those reported in Section 2.2.1 for the experiments with BST0.4. Therefore, we have estimated the sound velocity in OAT from the approximate equality of the acoustic impedances of the La_0.9_Sr_1.1_NiO_4_ and STO, $v_{OAT}\approx \left(\frac{\rho_{STO}}{\rho_{OAT}}\right)v_{STO}$. The parameters of the STO substrate$,\;\rho_{STO}≅5.1\;\mathrm{kg}/m^{3},\;v_{STO}≅8\;\mathrm{nm}/\mathrm{ps}$, are well known [50,51]. For the La_0.9_Sr_1.1_NiO_4_ OAT density estimate, we took the reported density of LaSrNiO_4_ [75], the composition of which is very close to the composition of our OAT, $\rho_{OAT}\approx \rho_{LSNO}≅6.4\;\mathrm{kg}/m^{3}$. With these assumptions, we estimated $\;v_{OAT}≅6.4\;\mathrm{nm}/\mathrm{ps}$ and, finally, $\tau_{a}^{theor}\approx 11-14$ ps. This estimate is very close to the experimentally revealed value $\tau_{a}^{exp}\approx \;$13 ps, indicating, in our opinion, that experimental result is reliable and that it is possible to use the measured duration of the CAP for the estimate of the green light penetration depth in La_0.9_Sr_1.1_NiO_4_, the relevant physical parameters of which are absent in the literature, $l_{\mathrm{La}0.9\mathrm{Sr}1.1\mathrm{NiO}4\;}^{green}\approx 83$ nm.

There are other, although less quantitative, indications that our estimates of the pump green light penetration depth are reliable. From [75], it is possible to estimate the specific heat of LSNO at room temperature $c\approx 9.1\cdot 10^{4}$ J/°K/m^3^. The thermal conductivity of LSNO measured in [75] is κ≈1.9 W/°K/m. Thus, the thermal diffusivity of LSNO is $D=\kappa /c\approx 2\cdot 10^{-5}\;m^{2}/s$. Assuming the same magnitude of thermal diffusivity in La_0.9_Sr_1.1_NiO_4_, it is possible to estimate the characteristic time of the temperature variation $\tau_{T}$ in the OAT due to heat transport in OAT after the laser excitation and the photo-excited charge carriers relaxation, $\tau_{T}\sim {\left(l_{OAT}^{green}\right)}^{2}/\left(\pi D\right)\approx \;$110 ps. This long time is correlated to our experimental observations of the slow variations of background in ΔR/R signals detected by UV probe in the temporal window of 40 ps duration around the time of CAP arrival at the free surface of BSTx, where the fitting of the theoretical model to the experimental results was accomplished in Section 3.1.

### 3.3. On the Brillouin Frequencies, Sound Velocities and Optical Refractive Indices of BSTx Library

The Brillouin frequency for a particular wavelength of probe light is one of the material fingerprints that provide opportunity to compare different materials or the materials of nominally same composition but that were synthesized/grown by different methods. From the physics point of view, the Brillouin frequency is the variation in the frequency of the probe light that could be caused by its interaction with sound. It depends on the sound velocity v and the optical refractive index *n* (Equation (1)) and can be used for the comparison of the different materials or as a contrast parameter in imaging of material spatial inhomogeneities [36,37,40], when the evaluation of v and *n* independently is either impossible or not required. Therefore, the revealed Brillouin frequencies (Figure 6f), sound velocities (Figure 6e), and refractive indices (Figure 6g) in the BSTx library are the fingerprints of the BSTx film grown by PLD at particular experimental conditions, such as oxygen pressure, substrate temperature, choice of a substrate for the deposition, optical scheme, etc. These fingerprints provide opportunity for the comparison of our tested Ba_1−x_Sr_x_TiO_3_ film with the films grown under different conditions and/or by other methods, as well as with the bulk materials of the same composition and either similar or different crystallinity. Below we compare the Brillouin frequencies, sound velocities and refractive indices of the BSTx films with the parameters of the bulk samples and of the films grown mostly by PLD, which were either determined by us in Section 2.2.2 or are reported in the literature, and we discuss these comparisons.

When approaching the STO edge of the BSTx library, the measured Brillouin frequencies at both probe wavelengths ($f_{B,\;BSTx}^{UV,STO}\leq 115\;\mathrm{GHz}\;\mathrm{and}\;f_{B,BSTx}^{green,STO}\leq 65\;\mathrm{GHz})\;$are lower than those measured by us in the STO substrate in Figure 4b ($f_{B,\;STO}^{UV}≅128\;\mathrm{GHz}\;\mathrm{and}\;f_{B,STO}^{green}≅72\;\mathrm{GHz})$ by about 10%. At the same time, the sound velocity at the STO edge is approaching the values $v_{BSTx}^{STO}\approx 8.1\;\mathrm{nm}/\mathrm{ps}$, which is in perfect correlation with multiple measurements of $v_{STO}\;$in bulk STO (see, for example [50]). Note that another frequently measured value for the velocity of the longitudinal waves in STO, i.e., $v_{STO}\approx 8.0\;\mathrm{nm}/\mathrm{ps}\;$[50,51], is well inside the uncertainty of our velocity measurements (Figure 6e). Therefore, the difference between the Brillouin frequencies at the STO edge of the library and the Brillouin frequencies in bulk STO is predominantly due to the difference in their refractive indices. When approaching the STO edge of the BSTx library, the measured refractive index (Figure 6f) at the UV probe wavelength ($n_{\;BSTx}^{UV,STO}\approx 2.5)\;$is lower than in bulk STO, measured by us in the STO substrate in Section 2.2.2. and reported in the literature [51,52,53,54,55] ($n_{\;STO}^{UV}\approx 2.9)$. A commonly accepted explanation for the difference in the optical refractive indices of the films prepared by PLD from those in bulk materials is the deviation from the ideal stoichiometry, which results in the increase of lattice parameters accompanied by the modification of the free charge carrier density, the variation in the impurity bands and shift of the charge transfer optical energies. The structural imperfections and oxygen vacancies in most cases call for a distinct reduction of the refractive index.

It is very difficult to produce STO films with stoichiometry comparable to STO bulk in terms of Sr/Ti ratio and oxygen content [76,77]. Cation off-stoichiometry (Sr or Ti vacancies) leads to an extended lattice parameter in STO epitaxial films, while O vacancies have little to no effect on the lattice parameter [76]. We observed a lattice extension on the STO side of the BSTx library, as can be seen in Figure 1c, with a *c* lattice parameter of about 3.92 Å vs. 3.905 Å for cubic bulk STO. This lattice extension ∆*c* of 0.015 Å is relatively limited compared to a maximum ∆*c* of 0.12 Å in [76] and should correspond to a very small cationic off-stoichiometry. As a laser fluence of 2 J/cm^2^ was used for deposition, we speculate that the STO film is Ti rich, as reported in [76] for fluence higher than 0.3 J/cm^2^. In STO perovskite structure, it is energetically unfavorable to accommodate interstitial Ti, so the most probable scenario for a Ti-rich film corresponds to Sr vacancies. The high oxygen pressure during post annealing should have reduced O vacancies to the minimum required for charge neutrality with the cations off-stoichiometry [78]. The very small deviation from stoichiometric STO in the STO side of our BSTx library is further confirmed by impedance spectroscopy measurement of the dielectric permittivity, with ε*_r_* = 304 (see Figure 1d for Sr = 100%) being very close to ε*_r_* = 310 found in bulk single crystal STO [79].

In reference [76] it is demonstrated that a Sr/Ti ratio change of 1% is sufficient to change the effective carrier density by orders of magnitude. The increase in the out-of-plane lattice parameter Δc was correlated in reference [76] to the cation non-stoichiometry, while the changes in electrical conductivity of the STO films were correlated to the expansion of the lattice. In accordance with [76], the lattice defects induced by small stoichiometry errors are responsible for the generally inferior electronic properties of complex oxide thin films when compared to equivalent bulk crystals. Conventional four-probe dc transport measurements revealed sheet resistance data which were consistent with the optical absorption measurements in oxygen-deficient STO films deposited at low oxygen pressures under various conditions [77]. The correlation between the characterization of charges in the materials by different measurement techniques operating at different frequencies of the electro-magnetic field are commonly expected. Thus, it is not surprising that our measurements of the optical refractive indices presented in Figure 6g reveal clear correlation to the 100 kHz dielectric permittivity measurements presented in Figure 1d on the films grown at high oxygen pressure, where the presence of oxygen vacancies is minimal. However, this correlation of the electrical and optical measurements in the BSTx library in combination with above-summarized results from reference [76] indicate that lower refractive indices measured by us at STO edge of the library were presumably caused by Sr/Ti ratio off-stoichiometry.

The observations at the opposite edge of the BSTx library are very different. When approaching the BTO edge of the film, the measured Brillouin frequencies at two probe wavelengths are $f_{B,\;BSTx}^{UV,BTO}\approx 75\;\mathrm{GHz}\;\mathrm{and}\;f_{B,BSTx}^{green,BTO}\approx 45\;\mathrm{GHz}$. The Brillouin frequency at our UV probe wavelength $f_{B,\;BTO}^{356\mathrm{nm}}$ has been never measured to our knowledge, while the Brillouin frequency for green light in BTO single crystals was reported in classical frequency-domain Brillouin scattering experiments ($f_{B,\;BTO}^{532\mathrm{nm}}\approx 53.5\;\mathrm{GHz}$ [80]). At the same time, the sound velocity measured by us at the BTO edge is approaching the value $v_{BSTx}^{BTO}\approx 4.9\;\mathrm{nm}/\mathrm{ps}$, which is at least 15% lower than the value reported for bulk BTO ($v_{BTO}\geq 6.2\;\mathrm{nm}/\mathrm{ps}$ [50]). So, slow propagation of acoustic waves in BTO was reported only for the polycrystalline ceramic samples synthesized by a solid phase reaction from high purity grade oxide and carbonates via calcination, homogenization and sintering at elevated temperatures [20]. Curiously, similar to our observations, the acoustic velocity in ceramics exhibits diminishing when x increases to 0.2–0.3, before definite growth for x approaching 0.4. When approaching the BTO edge of the BSTx library, the measured refractive indices at two probe wavelengths are $n_{\;BSTx}^{UV,BTO}\approx 2.67\;\mathrm{and}\;n_{BSTx}^{green,BTO}\approx 2.40$, respectively. The refractive index measured by us with green probe is practically identical to the earlier reported value $n_{BTO}^{535\mathrm{nm}}\approx 2.48$ [81]. In reference [81], the refractive index of BTO is documented down to 400 nm wavelengths, demonstrating a stable quasi-linear fast growth in UV region. Extrapolation of the dependence reported in [81] to our UV probe wavelength suggests $n_{BTO}^{356\mathrm{nm}}\approx 2.78$, close to our measured value and well inside our measurement uncertainty. Therefore, the refractive indices at the BTO edge of the BSTx library are practically identical to those in BTO melt-grown crystal [81]. With these refractive indices estimated from [81], the Brillouin frequencies in BTO, even using the lowest sound velocity of $v_{BTO}\approx 6.175\;\mathrm{nm}/\mathrm{ps}$ reported for bulk BTO in [50], are estimated as $f_{B,\;BTO}^{UV}≅96\;\mathrm{GHz}\;\mathrm{and}\;f_{B,BTO}^{green}≅57\;\mathrm{GHz}$. Note that the earlier-mentioned Brillouin frequency $f_{B,\;BTO}^{532\mathrm{nm}}\;$measured in [80] is just 6% below the estimated $f_{B,BTO}^{green}$, while the Brillouin frequencies $f_{B,\;BSTx}^{UV,BTO}\;\mathrm{and}\;f_{B,BSTx}^{green,BTO}\;$measured by us with UV and green probes are both lower by about 20%. Thus, in comparison with the STO edge of the BSTx film, the Brillouin frequencies at the BTO edge are also lower than in the bulk samples, but the reduction in the Brillouin frequencies are dominated not by the reduction of the refractive index but by the reduction of sound velocity.

The acoustic velocity of the longitudinal acoustic waves is controlled by the material longitudinal modulus *L* and the material density , $v=\sqrt{L/\rho }$. The deviation of the parameters of the elementary cell of BSTx film reported in Figure 1c from those in bulk BTO (see [82] and the references therein) is so small that the deviation of the cell volume from the one reported for bulk crystals is, in our estimates, smaller than 0.1%. The cell parameters in the film are larger than in bulk BTO. This deviation could lead to the diminishing of the material density and small variations of the material moduli, but overall could lead to hypothetical diminishing of the sound velocity which is negligible in comparison with that revealed by us experimentally. Comparison of the acoustic velocities at the BTO edge of the film with those measured in [20] provides insight that the diminished value of the sound velocity could be caused by the grain structure and/or porosity/defects in the Ba-rich BSTx films. Our FIB/STEM characterization of the BSTx film thickness revealed additional columnar growth of the films at the Ba-rich side of the film (at *Y* = 1 mm). Our observations of columnar growth are in correlation with experimental results on the dependence of the crystallinity of the BTO films deposited by PLD on the oxygen pressure (Figure 3 of [70]) obtained by FIB/TEM. In BTO films prepared by PLD on SRO film over STO substrate with increasing oxygen pressure, first, bombardment damage and grain boundaries of large grains were revealed at low pressure (5 mTorr), followed by formation of smaller grains in a fully dense microstructure at higher pressure (40 mTorr), and, finally, the growth of the columns separated by the gaps (evidencing not fully dense microstructure) at even higher pressure (200 mTorr). Therefore, although some deposition conditions in [70], such as the choice of the SRO instead of LSNO for the buffer layer and the temperature regime, are different from those in our experiments; the results in [70] support our observation of the columnar texture at our deposition pressure (75 mTorr). A rather common experience in this situation is that in polycrystalline/granular materials, the diminishing of the aggregate elastic moduli (due to the fact that grains boundaries are softer than the grains) overcompensates the diminishing of material density of a not fully dense microstructure relative to single crystals values and causes the diminishing of sound velocity.

Strong dependence of the film parameters on a variety of the conditions of the film deposition/growing, which is known from the numerous publications, including those referenced in the above discussion, is also supported by the comparison of our measurements presented in Section 2.2.2 and 2.2.3. There is about 18% difference between the Brillouin frequencies measured in the Ba_0.6_Sr_0.4_TiO_3_ (BST0.4) film ($f_{B,BST0.4}^{UV}=77\;\mathrm{GHz},\;$Figure 5d) and measured in the position corresponding to x=0.4 in the BSTx library ($f_{B,BSTx}^{UV}\left(x=0.4\right)=95\;\mathrm{GHz},$ Figure 6d). The most plausible explanation of this experimental observation is related to the fact that, in these samples, the films were deposited on the different structures, i.e., on SrTiO_3_/SrRuO_3_ (STO/SRO) and SrTiO_3_/La0_.9_Sr_1.1_NiO_4_ (STO/LSNO), respectively. The dependence of the refractive index of the films prepared by PLD on the choice of the substrate is well documented in the literature. For example, the refractive indices of the Ba_0.8_Sr_0.2_TiO_3_ (BST0.2) films deposited on Si/SiO_2_/Ti/Pt, Si/SrRuO_3_ and Si were different at the 356 nm probe optical wavelength of our interest up to 13% (Figure 4c in [68]). The sound velocities in thin films also depend on the choice of the structure for their deposition. Unfortunately, we failed to find in the literature the examples for the films deposited with pulsed lasers on different substrates. However, the examples for the thin films prepared by other techniques are rather common. For example, in [43] the difference in the sound velocities of (Bi_1−x_Pr_x_)(Fe_0.95_Mn_0.05_)O_3_ films deposited by sol-gel method on Si and LaAlO_3_ (LAO) substrates is up to 8% even for the x = 0.2 composition, which is sufficiently far from the structural phase transition around x = 0.12–0.16, where the differences in sound velocities are much more significant. The dependences of the optical refractive index and of the sound velocity in the thin films on the structure/substrate chosen for their deposition could both contribute to the differences in the Brillouin frequencies in the thin films deposited on different structures/substrates. Thus, from the physics point of view, the difference between the Brillouin frequencies measured in the Ba_0.6_Sr_0.4_TiO_3_ (BST0.4) film and measured in the position corresponding to x = 0.4 in the BSTx library is caused by the difference in the lattice parameters of the SRO and LSNO and the difference of both from the lattice parameter of Ba_0.6_Sr_0.4_TiO_3_, which results in the difference of the relaxation processes in the epitaxial deposited films and, finally, in different optical refractive indices and acoustic velocities of these films.

We conclude this discussion with the statement that the suggested plausible explanations for the magnitudes of $f_{B}$, v and n at STO and BTO edges of the BSTx material library as well as the correlation between the electrical measurements of the permittivity in Figure 1d and the optical measurements of the refractive index in Figure 6g provide confidence that our measurements of some earlier unknown parameters ($f_{B}$, v and n) in the composition range 0 < x < 1 of BSTx are reliable.

## 4. Conclusions and Perspectives

We demonstrated an application of PLU in combination with XRD, EDS, EPMA, SEM and AFM for the measurements of the dependencies on x of the optical refractive index n and of the sound velocity v in an epitaxially grown thin film BSTx library. A continuous composition spread thin film of BSTx was deposited over an opaque optoacoustic thin film transducer, required for the application of PLU in transparent films/coatings, by combinatorial pulsed laser deposition (CPLD) on a single substrate. The complete BSTx solid solution was thus studied by PLU on a unique sample. This way, the acoustical and optical parameters, extracted from the acoustically induced changes in the ultrafast pump-probe transient optical reflectivity as a function of local chemical composition, were preserved from run-to-run variation that might occur when successive samples with different chemical compositions are sequentially prepared.

The lateral composition of the BSTx library was revealed by XRD, EDS and EPMA. The acoustical (sound velocity) and optical (refractive index) parameters of BSTx for all values of composition parameter x were determined for the first time. To determine the sound velocities, $v=d/\tau_{d}$, the thickness *d* of the BSTx films was measured by SEM, while the sound propagation time across the film $\tau_{d}\;$was obtained by fitting the experimentally revealed dynamics of acoustically induced film thickness variations by the theoretical model. Refractive indices *n* were then determined due to the obtained knowledge of sound velocities by measuring the frequency of the Brillouin oscillation in PLU signals. The sound velocities in the epitaxially grown BSTx film at its Sr-rich edge are comparable to earlier reported ones in the commercially available STO substrates. The sound velocity in the epitaxially grown BSTx film at its Ba-rich edge is inside a rather broad range of values reported earlier under the different conditions of the BST films and crystals growth. Generally speaking, our measurements revealed that the sound velocities of the BSTx epitaxially grown films are similar to those measured in bulk crystals. The refractive indices of the epitaxially-grown BSTx films are systematically lower than the values measured in bulk crystals, presumably caused by Sr/Ti ratio off-stoichiometry, as discussed in detail in Section 3.3.

We applied new protocol for modeling and fitting the PLU signals, which accounts for the significant roughness of the film surface revealed by AFM measurements. The fitting of the PLU signals suggested the magnitudes of surface roughness in good correlation with AFM measurements, indicating a possible way for the characterization of surface roughness by PLU alone. The evaluation of the surface roughness by PLU could be important for the characterization of the individual films/layers that could be used in the design of other optoacoustic experiments, for example when composing the multi-layered structures, i.e., when growing photonic, phononic or phoxonic superlattices/crystals. In this case, PLU measurements of the roughness would provide access to the quantitative prediction of the CAPs propagation in these structures, while the measurements by AFM could support only qualitative predictions.

The high-throughput characterization of the optical and acoustical properties was achieved via application for PLU of a custom ASOPS ultrafast lasers system, confirming that PLU can be a rapid and nondestructive characterization technique for analyzing compositionally graded films. Although the ASOPS-based PLU measurement took 3 min and 20 s per point in our case, we discussed in Section 2.2.1 how this acquisition time could be reduced and we would like to remind the readers here that the most important support for the high throughput and rapidness claims of the PLU technique is its uniqueness in providing opportunity to determine simultaneously optical and acoustical parameters (and, potentially, acousto-optic/photo-elastic parameters) of the films.

Of course, the crucial contribution to the fast characterization of thin films in our above-reported experiments is due to chosen technique of the epitaxial film deposition. The combinatorial PLD can be directly compared to conventional PLD or sputtering deposition techniques. The deposition time for one composition spread library is about 20% longer than for a sample with uniform composition, about a few hours in total. Calibration runs are necessary to estimate deposition rate for each target used in combinatorial PLD. On one combinatorial library, there is a continuum of compositions which can be measured. In this article, 19 compositions are probed, which would have required the synthesis of 19 different samples with conventional deposition techniques, with the risk of run-to-run variations. Furthermore, measuring locally a unique library sample with different characterization techniques having a small probing area only requires X,Y stage to move from one location to another, i.e., from one composition to the next, and can be automatized. This is much faster than mounting/fine-tuning/dismounting several samples. Depending on the number of the compositions probed and of the characterization techniques used, the time scale can be shortened by weeks or months using this high-throughput synthesis.

Engineering of phononic resonances in ferroelectric structures to realize novel multifunctional devices is one of the recent directions in nanophononics [49,83,84,85], starting from the concept and design of acoustic Bragg mirrors and cavities made of multilayers of piezoelectric oxides (BaTiO_3_∕SrTiO_3_ superlattices on SrTiO_3_ substrates) for potential applications in electronic and optical terahertz modulators [83]. The strong coupling between sound, charge and light in these multifunctional nanoscale ferroelectrics was demonstrated [84], as well as the application of BaTiO3*/*SrTiO3 cavities for enhancement and inhibition of coherent phonon emission [49]. In [85], the confinement of acoustic waves in the 100-GHz frequency range in a phonon nanocavity, as well as the time and spatial beatings resulting from the coupling of two different hybrid nanocavities forming an acoustic molecule, was experimentally demonstrated, introducing ferroelectric cavity systems as a new realm for the study of complex wave localization phenomena at the nanoscale. We hope that the first measurements of the acoustical and optical properties of the epitaxially-grown Ba_1−x_Sr_x_TiO_3_ (0 ≤x≤1) by picosecond laser ultrasonics technique, in conjugation with X-ray diffraction, energy dispersive spectroscopy, electron probe microanalysis, scanning electron and acoustic force microscopies, presented by us will provide the parameters for more extended predictive design of the phononic, photonic and phoxonic mirrors and cavities with superior properties/functionalities.

## Data Availability

The data presented in this study are available from the corresponding author upon reasonable request.
